# Challenges in managing recurrent luminal A breast cancer: a case study

**DOI:** 10.3332/ecancer.2025.2048

**Published:** 2025-11-26

**Authors:** Md Foorquan Hashmi, Fiza Khan, Elen Baloyan, Liana Safaryan, Davit Zohrabyan, Gevorg Tamamyan, Samvel Bardakhchyan

**Affiliations:** 1 Yerevan State Medical University after Mkhitar Heratsi, Yerevan 0025, Armenia; 2 Immune Oncology Research Institute, Yerevan 0014, Armenia; 3 Yeolyan Hematology and Oncology Center, Yerevan 0014, Armenia

**Keywords:** breast cancer, luminal A, mastectomy, hereditary angioedema, young age

## Abstract

Breast cancer, a leading cause of cancer-related mortality in women worldwide, exhibits diverse molecular subtypes with varying prognostic implications. Luminal A breast cancer, characterised by estrogen receptor positivity, progesterone receptor positivity and human epidermal growth factor receptor 2 negativity, typically has a favourable prognosis. However, we present a case of a 35-year-old female diagnosed with Luminal A breast cancer who experienced multiple recurrences and succumbed to the disease within 2 years of diagnosis. Despite initial treatment modalities, including surgery, chemotherapy and hormonal therapy, the patient faced relentless disease progression, highlighting the atypical clinical course observed in younger individuals with Luminal A breast cancer. This case underscores the discrepancy in prognosis between younger and older patients with Luminal A breast cancer, emphasising the need for tailored treatment approaches to address the challenges associated with disease recurrence and aggressiveness in younger populations. Further research is warranted to explain the underlying mechanisms driving the aggressive behaviour of Luminal A breast cancer in young patients and to develop targeted therapeutic strategies to improve outcomes in this population.

## Introduction

Breast cancer stands as the leading cancer diagnosis among women, representing over 10% of new cancer cases annually. Globally, it ranks as the second most prevalent cause of cancer-related deaths in women [1,2]. In 2020, there were 2.3 million women diagnosed with breast cancer and 685,000 deaths globally [1]. Certain factors increase the risk of breast cancer, including increasing age, obesity, harmful use of alcohol, family history of breast cancer, history of radiation exposure, reproductive history (such as age at which menstrual periods began and age at first pregnancy), tobacco use and postmenopausal hormone therapy [1].

According to the PAM50 signature, breast cancer has been classified into five molecular subtypes: Luminal A (estrogen receptor (ER)+ and progesterone receptor (PR)+, human epidermal growth factor receptor 2 (HER2)-, low levels of Ki-67), Luminal B (ER+, HER2- and either Ki-67 high or PR low), Luminal B-like (ER+, HER2+, any Ki-67 and any PR), HER2-enriched (ER-, PR- and HER2+), triple negative or basal-like (ER−, PR- and HER2-) [3].

Breast cancer typically occurs more frequently in individuals aged 50 years or older. However, being diagnosed at a younger age has been linked to a poorer prognosis in breast cancer, indicating a tendency towards more aggressive disease [4].

Diagnosing breast cancer involves various methods of screening, including clinical breast examination, breast self-examination and breast imaging techniques such as mammography, ultrasonography, magnetic resonance imaging (MRI) and digital breast tomosynthesis [5]. Among these, mammography and ultrasound are primary imaging techniques utilised for screening, diagnosing breast cancer, determining its stage, evaluating treatment response and monitoring post-treatment follow-up. These screening methods play a crucial role in identifying breast cancer early, allowing for prompt intervention and improved outcomes.

In the treatment landscape, particularly for luminal A breast cancer, hormone therapy emerges as a frequently preferred option. Early-stage breast cancer management typically begins with surgery followed by adjuvant hormone therapy, with or without chemotherapy, depending on lymph node involvement and menopausal status [5].

In luminal breast cancer, biomarkers play a crucial role in diagnosis, prognosis and determining treatment options, contributing significantly to enhanced patient outcomes [6]. In the advanced stage, three different categories of targeted medications (novel treatments)—mTOR inhibitors, CDK 4/6 inhibitors and PI3K inhibitors—are authorised for treatment. CDK 4/6 inhibitors have significantly enhanced outcomes, fundamentally altering the progression of advanced luminal breast cancer [5].

Luminal A tumours are characterised by being low-grade and slow growing, whereas luminal B tumours exhibit higher aggressiveness with a faster proliferative rate [7]. While luminal A cancers typically pose a low risk of local or regional recurrence, they may demonstrate a tendency to metastasize in later stages, often occurring more than 5 years after symptom onset, with lymph nodes and bones being common sites of metastasis [8]. According to data from the Surveillance, Epidemiology and End Results (SEERs) database, the 5-year survival rate for luminal A breast cancer is 94.4%, compared to 90.7% for luminal B tumours [2].

This case report presents the clinical course of a 35-year-old female diagnosed with breast cancer. Despite initial treatment modalities, the patient experienced recurrence, leading to a series of surgeries and multiple lines of chemotherapy. The case highlights the challenges in managing recurrent breast cancer.

## Case presentation

The patient, a 35-year-old female of 45 kg, and a parity of two children from three pregnancies, with disease of hereditary angioedema, presented with mastitis of the left breast during breastfeeding. Imaging studies revealed a 16 × 14 mm mass in the upper lateral quadrant of the left breast (BI-RADS 4) and axillary lymphadenopathy. Core biopsy was not informative. The patient underwent surgery. Subsequent sectoral resection with postoperative histological and immunohistochemistry (IHC) confirmed invasive breast cancer, no special type, with sentinel lymph node involvement: G2, pT1c pN1a (sn2/2) cM0, ER7+, PR7+, Her2=, Ki67 15-18%, Luminal type A, IA pathological prognostic stage. Pretreatment neutrophil-to-lymphocyte ratio was 2, within the normal range. Tumour-infiltrating lymphocytes were not assessed. Computed tomography (CT) scans showed no distant metastases. Breast cancer susceptibility proteins 1 and 2 were not checked. Because of positive lymph nodes and premenopausal status, adjuvant chemotherapy with Epirubicin + Cyclophosphamide was initiated.

**Figure 1.**
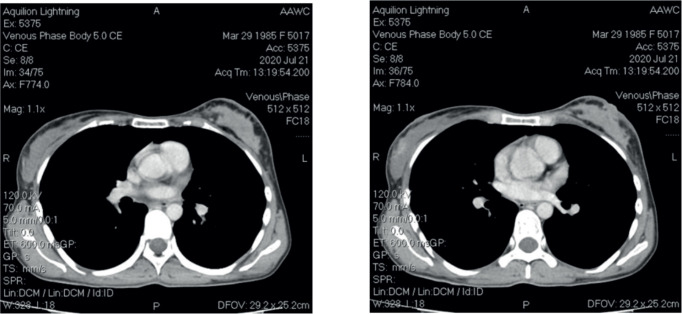
CT scan images.

After four cycles, a mammogram revealed local recurrence in the left breast and a new lesion in the right breast (BI-RADS-3) with axillary lymphadenopathy on both sides. CT scan demonstrated total disorganisation of the left breast with skin thickening and a new suspicious lesion in the right breast (Figure 1). No distant metastases were observed.

A second surgery, 5 months after the first surgery, included left mastectomy with axillary lymph node dissection and sectoral resection of the right breast (intraoperative histology was invasive cancer). Postoperative pathology of left breast was yG2 ypT3 ypN2a (9/10) L1 V0 Pn1, R1, ER+, PR+, Her2= and for right breast was G2 pTx pN2a (5/12) cM0 L1 V0 Pn1 R0. ER+, PR+, Her2 =, Ki67 50-60%, IB pathological prognostic stage. Following the surgery, hormonal therapy was initiated with Tamoxifen and gonadotropin-releasing hormone agonist Goserelin.

However, 2 months post-surgery, the patient identified subcutaneous nodules near the postmastectomy scar on the left chest wall. Mammography confirmed local recurrence in the left breast on the postmastectomy scar and revealed a new suspicious lesion in the right breast. A subsequent CT scan demonstrated no distant metastases. In response to this recurrence, chemotherapy was started with Carboplatin + Paclitaxel regimen. Remarkably, after completing four cycles of chemotherapy, the patient exhibited a partial response, indicating a positive therapeutic outcome. A third surgery involved resection of the left chest wall recurrent tumour and right mastectomy. Postoperative pathology showed recurrent tumours in both breasts. It showed a partial response to chemotherapy. Subsequently, chemotherapy with Carboplatin + Paclitaxel was continued.

**Figure 2.**
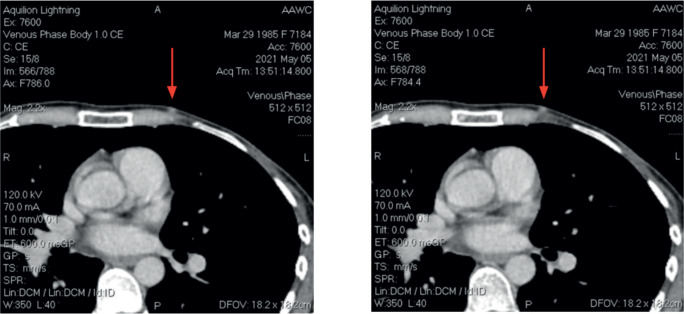
CT Images at third recurrence.

After the second postoperative cycle, the patient palpated three new subcutaneous nodules on the left chest wall, confirmed by CT scan (Figure 2). No distant metastases were identified. A fourth surgery focused on the removal of subcutaneous lesions. Postoperative pathology confirmed breast cancer in all three specimens. The tumour board recommended postoperative radiotherapy (RT) and hormonal therapy with AI ± CDK4/6 inhibitors. The patients went abroad to Germany, where new left axillary lymphadenopathy was found. The patient had another surgery: left axillary lymph node dissection. Afterward, she received RT combined with Capecitabine and two cycles of Capecitabine after RT. However, follow up with a CT scan revealed recurrence with new lesions in the liver. Treatment was adjusted to Ribociclib plus Fulvestrant, which she received for 5 months without a major response.

Subsequently, the patient developed coagulopathy, fourth-grade thrombocytopenia and liver failure. Palliative care was advised, and she passed away – 2 years after the initial diagnosis.

## Discussion

Luminal A hormone receptor-positive (HR+/HER2-) stands as the predominant subtype in breast cancer, making up approximately 68% of cases as reported by the National Cancer Institute's SEER database [2]. In contrast to other subtypes, luminal A progresses at a slower rate, demonstrates lower aggressiveness and typically has better outcomes.

One of the notable observations in this case is the young age of the patient at diagnosis. While breast cancer is more prevalent in older women, younger age at onset is considered an adverse prognostic factor, often associated with more aggressive disease behaviour [9]. Recent studies have examined age at diagnosis as a key prognostic factor across molecular subtypes [9]. Moreover, younger patients have a higher likelihood of developing bilateral breast cancer [10]. Consistent with this, our case involved bilateral breast affection in a younger patient.

MRI has been shown to have higher sensitivity, but lower specificity, compared to mammography in detecting breast cancer. For instance, Aristokli *et al* [11] reported MRI sensitivity at 94.6% (range: 85.7%–100%) and specificity at 74.2% (range: 25%–100%), whereas mammography showed sensitivity of 54.5% (range: 27%–86.8%) and specificity of 85.5% (range: 62.9%–98.8%). In this case, mammography performed due to mastitis revealed a suspicious lesion in the left breast along with axillary lymphadenopathy. MRI was not performed due to financial limitations and because the mammographic findings did not suggest the need for further clarification.

For early-stage breast cancer, the primary treatment involves surgery followed by adjuvant hormone therapy, with or without chemotherapy, depending on the involvement of lymph nodes and the patient's menopausal status. However, despite existing controversies regarding the use of chemotherapy in luminal A, lymph node-positive breast cancer [5], its application is supported due to the high-risk nature of lymph node positivity. Chemotherapy can provide benefits to breast cancer patients with positive lymph nodes, especially in the premenopausal subgroup [5]. According to Coates *et al* [12], chemotherapy is recommended for luminal A breast cancer only when there are four or more positive lymph nodes. But as per the guidelines from the National Comprehensive Cancer Network, chemotherapy is recommended for premenopausal patients with luminal A breast cancer who have positive lymph nodes, irrespective of the node count [5]. The patient initially received a combination of Epirubicin and Cyclophosphamide as chemotherapy, but developed disease recurrence.

Typically, for HR+ and HER2-negative tumours, which include both luminal A and B subtypes, systemic adjuvant therapy relies on hormone therapy, with variations depending on whether the patient is pre- or post-menopausal. Luminal A subtype generally carries a favourable prognosis, and the systemic adjuvant therapy commonly involves hormone therapy alone [4]. However, a common challenge arises as tumours often develop resistance to this treatment modality as the disease progresses. Mechanisms underlying hormone therapy resistance encompass various aberrations within the ER/PgR pathway, including deregulation of ER expression, alterations in co-activators and co-repressors, genomic and epigenetic changes in ERS1, expression of truncated ER-isoforms, post-translational modifications, heightened receptor tyrosine kinase signaling and disrupted cell cycle regulation [13]. Despite initial treatment with Tamoxifen, the patient exhibited no response and subsequently developed local recurrence, indicative of resistance to hormone therapy.

Novel treatment modalities, including targeted therapies toward PIK3CA, breast cancer susceptibility protein 1/2 (BRCA1/2), CDK4/6 and mTOR pathways, have shown promise in overcoming hormone therapy resistance in luminal breast cancers. A study by Santarpia *et al* [14] identified PIK3CA as the gene most mutated in IHC-based ER+/luminal cancers. According to data from The Cancer Genome Atlas, the PIK3CA E545K mutation is predominantly present in the luminal A subtype [14], and agents such as alpelisib and capivasertib with fulvestrant are FDA-approved for such cases [15]. BRCA1, BRCA2 and partner and localiser of BRCA2 are implicated in the repair process of DNA double-strand breaks [6]. The FDA has granted approval for the therapeutic use of two PARP inhibitors, namely olaparib and talazoparib, in breast cancers with BRCA1/2 mutations [15]. CDK4/6 inhibition has emerged as the most effective approach [15]. The three drugs currently approved are Palbociclib, Abemaciclib and Ribociclib, have demonstrated notable efficacy leading to considerably prolonged progression-free survival (PFS) and even overall survival [16].

In the context of metastatic breast cancer, the efficacy of ribociclib combined with hormone therapy was investigated specifically in a first-line trial involving exclusively pre/perimenopausal participants (MONALEESA-7), showing significantly prolonged PFS and overall survival compared to AI treatment alone [17]. In our case, Ribocilib was used in the later course of the disease but did not show significant efficacy and the patient developed liver failure, leading to death.

However, in low-resource settings, several barriers limit the practical application of these advances. Financial constraints often preclude molecular profiling (e.g., PIK3CA, BRCA1/2 and NGS), and even when such mutations are identified, high drug costs and limited availability—particularly of CDK4/6 inhibitors can prevent their use. This was the case for our patient, where lack of access to comprehensive genomic testing and certain targeted agents likely restricted treatment options and hindered the ability to explore resistance mechanisms.

**Figure 3.**
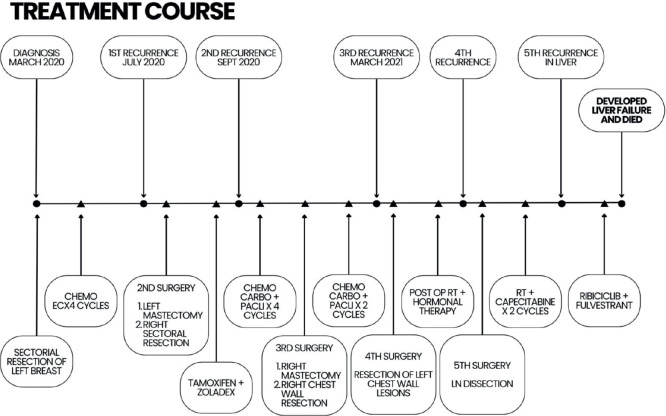
Patient’s complete treatment course.

Breast cancer patients typically receive an early diagnosis in 79%–87% of cases, falling into stages I or II, while 13%–21% are diagnosed at a later stage (stages III or IV). Around 7% of breast cancer patients already have metastases at the time of diagnosis [18]. Approximately 15% of patients will experience a loco-regional relapse, where the tumour is found in the breast and/or regional lymph nodes, within the subsequent 5 years [2]. In our case, the patient had multiple recurrences in a span of 2 years only which is not typical for Luminal A type. Initially, sectoral resections with lymph node dissections were done, but eventually, due to recurrences and new lesions, a radical mastectomy was performed. Adjuvant therapies with RT, chemotherapy and hormonal therapy were also employed. Additionally, early recurrence in the Luminal A subtype has been linked to poor overall survival [19], as observed in our case, where the patient experienced multiple recurrences (Figure 3).

Luminal A breast cancer has a 5-year survival rate of 94.4%, while luminal B’s rate is 90.7%, HER2-enriched is 84.8% and triple-negative is 77.1%, according to SEER [2].

Subtypes identified through gene expression and IHC serve as standalone indicators of survival and recurrence risk. This holds true even during the 5-year clinical follow-up period post-diagnosis, highlighting luminal breast cancer's favourable prognosis and triple-negative disease's unfavourable outlook [20]. Despite the patient's diagnosis of Luminal A breast cancer, the anticipated outcomes were not achieved and the patient succumbed to the disease 2 years after the initial diagnosis.

## Conclusion

This case report underlines the atypical clinical course observed in a 35-year-old female diagnosed with Luminal A breast cancer. Despite the generally favourable prognosis associated with Luminal A subtype, this patient experienced multiple recurrences and ultimately succumbed to the disease within 2 years of diagnosis. The findings highlight the discrepancy in prognosis between younger and older patients with Luminal A breast cancer, indicating a heightened risk of adverse outcomes in younger individuals. The case underscores the importance of considering age as a prognostic factor and the need for tailored treatment approaches in younger breast cancer patients to improve outcomes and address the challenges associated with disease recurrence and aggressiveness. Furthermore, it emphasises the necessity for further research to elucidate the underlying mechanisms driving the aggressive behaviour of Luminal A breast cancer and to develop targeted therapeutic strategies to address these challenges effectively.

## Authorship criteria

We, the undersigned authors, hereby confirm that:

- Each author has made a substantial contribution to the conception, design, acquisition, analysis or interpretation of data and to the drafting or critical revision of the manuscript.
- Each author has approved the final version of the manuscript submitted to ecancermedicalscience.
- Each author agrees to be accountable for all aspects of the work and to ensure that questions related to the accuracy or integrity of any part of the work are appropriately investigated and resolved.

## Conflicts of interest

None.

## Funding

None.

## Originality and ethics

We confirm that the manuscript is original, has not been published previously, and is not under consideration elsewhere.

We further confirm that ethical approval and informed consent were obtained.

## Author contributions

- Md Foorquan Hashmi: Literature Review, Manuscript Writing, Data Collection.
- Fiza Khan: Literature Review, Manuscript Writing, Administrative Support.
- Elen Baloyan: Patient Care & Clinical Management, Data Collection, Critical Revision.
- Liana Safaryan: Patient Care & Clinical Management, Data Collection, Literature Review.
- Davit Zohrabyan: Patient Care & Clinical Management, Data Collection, Literature Review.
- Gevorg Tamamyan: Literature Review, Conceptualisation, Supervision, Ethics & Consent.
- Samvel Bardakhchyan: Literature Review, Idea Generation, Critical Revision, Final Approval.
